# Tear biomarkers in latanoprost and bimatoprost treated eyes

**DOI:** 10.1371/journal.pone.0201740

**Published:** 2018-08-06

**Authors:** Shweta Reddy, Prity Sahay, Debananda Padhy, Sarada Sarangi, Mrutyunjay Suar, Rahul Modak, Aparna Rao

**Affiliations:** 1 Hyderabad Eye Research Foundation (HERF), L.V. Prasad Eye Institute, Bhubaneswar, India; 2 School of Biotechnology, KIIT University, Bhubaneswar, India; Boston University School of Medicine, UNITED STATES

## Abstract

**Purpose:**

Prostaglandin analogues (PGA’s) are the mainstay and first line of treatment in current glaucoma practise. Though latanoprost and bimatoprost are the most commonly used PGA’s with minimal side effects at lower concentrations like bimaotoprost 0.01%, direct comparison of their cytokine/MMP profile in tears has not been evaluated earlier. The study intends to ascribe PGA to the upregulation of MMPs, Cytokines and Chemokines mediating varied pathways to result in side effects of the drugs.

**Methods:**

Tear sample collection was done from outer canthus of 30 eyes of 30 patients (primary open angle glaucoma (n = 26 and n’ = 20), normal tension glaucoma (n = 4 and n’ = 10), in latanoprost (n) 0.005% and bimatoprost (n’) 0.01% group respectively, with a mean age of 62±10.5 years) on >6 months of PGA use using Tear flo^TM^ Schirmer filter strip. Tear samples from 30 eyes of 30 cataract patients without drug treatment were used as the control. Gelatinolytic activity of MMP-9 and MMP-2 were examined by substrate gelatine zymography MMP-1 and TIMP-1 concentrations from tears samples with PGAs were evaluated by ELISA while cytokine concentration in the eluted tears was evaluated using a convenient bioplex kit assay (Milliplex MAP kit, HCYTMAG-60K-PX41, Millipore, Massachusetts, United States). The mean duration of use of PGA in both groups did not differ significantly (median 1.3 years in bimatoprost and 1.1 years in latanoprost eyes, p = 0.6).

**Results:**

The tear MMP-9 expression was higher in eyes receiving latanoprost while the MMP-2 expression was higher in eyes receiving bimatoprost with MMP1 protein levels being higher in the former. Latanoprost treated eyes had marginally elevated tear cytokines involved in tissue remodelling while bimatoprost eyes showed elevated cytokines regulating allergic pathways.

**Conclusion:**

Differential cytokine and MMP expression indicates differential signalling pathways mediating different cellular effects (evident as clinical and side effects) with the two drugs which can be explored further.

## Introduction

Topical prostaglandin analogues (PGA) have become first-line therapy in medicinal management of current glaucoma practice.[1,2] The most commonly available PGA used globally include latanoprost (ester prodrug of PGF_2α_) and bimatoprost (amide prodrug of 17-phenyl-PGF_2α_)[2]. The PGA affords long term diurnal control of intraocular pressure (IOP) and halt visual field progression as compared to other ocular anti-hypertensives.[2,3–6] Though the systemic side effects of PGA are rare, ocular side effects are very frequent which have prompted the search for better drugs with better safety profiles on long term use. The side effects with PGA include conjunctival hyperemia which is the most frequent complaint, elongation and darkening of eyelashes and periocular skin.[3] Several rare vision threatening complications also include iris cysts, cystoid macular edema, anterior uveitis and reactivation of herpes simplex keratitis. While side effects of PGA may attribute to the preservatives, several are induced by upregulation of the MMPs and cytokines by the PGA themselves. The PGAs are known to induce MMPs expression in kertaocytes in-vitro and also in-vivo in the conjunctiva. These drugs have far superior efficacy in terms of IOP reduction by improving aqueous drainage via the uveoscleral pathway an also additionally work by remodelling the extracellular matrix (ECM).[1,3–7]

ECM homeostasis in the eye involves regulation of MMPs and regulation of the balance between MMPs and TIMPs. Previous studies showed that PGA induces expression of MMP-1, −2, −3, −9, and −17 and TIMP-1 and −2 in the human ciliary body.[6,7] Dysregulated ECM homeostasis tightly balanced and regulated by MMPs were suggested to be responsible for fibrotic ocular diseases including glaucoma. In glaucoma, altered ECM homeostasis in the trabecular meshwork leads to decreased degradation of ECM causing obstruction of aqueous outflow pathways. Therefore PGA acting via ECM regulation and MMPs assumes utmost importance in glaucoma responsible for more efficient IOP control. Inflammatory side effects in the eye like hyperemia and uveitis due to chronic use of ocular anti-hypertensives like PGAs are also known to be induced due to increased MMP activation. Interestingly, the doses and preservatives also have been therefore been modified as an attempt to reduces these side effects at the ocular surface and conjunctiva.[8–10] Bimatoprost 0.01% is reported to reduce the incidence of conjunctival hyperemia as compared to bimatoprost 0.03% while latanoprost is reported to have lesser side effects as compared to the former molecule.[10,11,12] Yet, a direct comparison of tear MMP profiles in patients receiving bimatoprost 0.01 and latanoprost 0.005% has not been studied earlier.

## Materials and methods

This observational study included glaucoma and cataract patients attending eye care services at L.V Prasad Eye Institute, Bhubaneswar and included all glaucoma patients seen at the glaucoma service during the period of January 2015 to December 2015 who were on either Latanoprost 0.005%, Xalatan, or Bimatoprost 0.01%, Lumigan, for more than 6 months. The study patients were selected after screening from a larger study involving adult primary glaucoma (defined as patients with open angles on gonioscopy, raised IOP >21mm Hg and optic disc/visual field changes consistent with diagnosis of glaucoma) which was approved by the Institutional review board (IRB) of LV Prasad Eye Institute, MTC campus, Bhuabaneswar and adhered to the tenets of the Declaration of Helsinki. An informed written consent was obtained from each subject before any ocular examination or procedure as institutional protocol. The patients with any secondary forms of glaucoma (like traumatic, uveitic, steroid glaucoma, neovascular), previous surgery, dry eye, history of contact lens wear, history of inflammatory or allergic disorders, use of other medications, other systemic or ocular co morbidities like (corneal opacity, corneal trauma, ulcer, diabetic retinopathy, venous occlusive diseases), were excluded from the study. Patients on other medications apart from those described above or using PGA of other manufacturers were also excluded. Patients with cataract with no other systemic or ocular associations were included as controls in the study. Each subject underwent detailed ophthalmic examination including slit lamp examination, refraction, fundus biomicroscopy, Humphrey visual fields, Schirmer’s test and tear sample collection.

### Tear collection

Tear sample collection was done from outer canthus of patients using Tear flo^TM^ Schirmer filter strip. The tear strips were placed in the inferior fornix for 5 minutes under aseptic conditions following which the Schirmer strip containing tear sample was stored in -20 degree until further analysis.

The Schirmer strips were placed in 200ul Protein Extraction Buffer (0.5 M NaCl and 0.5% Tween 20) for 2–3 hours at room temperature on a rocker (Tarsons Products, West Bengal, India) followed by centrifugation (Eppendorf, Hamburg, Germany) at 15000 rcf for 30 seconds. Eluted protein was precipitated using pre-chilled acetone. The protein amount was quantified using Bradford assay (Bio-Rad Protein Assay Dye Reagent-1X; Bio-Rad Laboratories, Hercules, CA, USA). A bio-spectrophotometer (Eppendorf) was used to read the absorbance at 595 nm with the results reported in μg/μl. An average estimate of total protein eluted from tears is 132 μg/μl, out of which we load 50 μg/μl sample per well.

### MMPs activity by gelatine zymography

Gelatinolytic activity of MMP-9 and MMP-2 were examined by substrate gelatine zymography. Equal amount of proteins obtained from tear samples of patients were separated on 10% SDS-PAGE gels containing 0.1% gelatin. The gels were washed twice with an interval of 1hour in 2.5% tritonX-100 washing buffer and then incubated in incubation buffer containing 50mM Tris-HCl, 10mM CaCl2, 1μM ZnCl_2_ and 200mM NaCl, pH 7.5 at 37°C for 18–20 hrs. Gels were stained with coomassie solution (0.05% coomassie brilliant blue R-250, in 40% methanol and 10% acetic acid) and partially destained with destaining solution (20% methanol and 10% acetic acid) to visualize clear zone of gelatin lysis against blue background stain indicating the presence of MMPs. The zymographic gels were imaged and lysis zones in every lane analyzed using image J software (http://imagej.nih.gov/ij/; provided in the public domain by the National Institutes of Health, Bethesda, MD, USA) to obtain band intensity with metalloproteinase 2 and 9 activity expressed in arbitrary units (A.U). Since sample storage for a long time would results in protein degradation and variable results, we analysed the gelatinolytic activity of samples within 2 days of collection.

### Enzyme linked Immunosorbent Assay (ELISA)

MMP-1 and TIMP-1 concentrations from tears samples with PGAs were determined with colorimetric immunoassays performed according to the instructions of the manufacturers (ThermoScientific, Massachusetts, United States). Tear samples were pooled using 10ul tears sample from each patient to make a total of 100ul sample. In brief, concentrations of MMP-1 (EHMMP1, sensitivity 8pg/ml) and TIMP-1 (KHC1491, sensitivity <1ng/ml) in 100ul of pooled tears sample was determined by incubating it overnight in plate coated with monoclonal anti-MMP-1 antibody or anti-TIMP-1 with gentle shaking at 4°C. Plate was washed with 1x wash buffer (PBS with 0.033% Tween 20) and incubated with 1X biotinylated secondary antibody for an hour at room temperature (RT). After washing again with wash buffer 100ul of streptavidin horseradish peroxidase was added into each well and incubated for 45 mints at RT. To visualize colour change 100ul of colour reagent (3,3’,5,5’-tetramethylbenzidine) was dispensed into each well and incubated for 30 min at RT in the dark followed by addition of 50ul of 0.2M sulfuric acid. Quantification of analytes was done by measuring the absorbance on an ELISA plate reader (Epoch 2, BioTek Instruments, Winooski, United states) at 450nm.

### Multiplex cytokines analysis

Concentration of cytokines in the eluted tears was evaluated using a convenient bioplex kit assay (Milliplex MAP kit, Hcytmag-60k-px41, Millipore, Massachusetts, United States). Pooled tear samples from 20 patients of the total cohort in each group were used in a multiplex bead assay running 25μl volume for 41 different cytokines available in pre mixed beads. Beads were briefly sonicated for 30 second and then vortexed for 1 minute. The cytokines included in this kit were as follows; Soluble CD40-ligand(Scd40L), Epidermal Growth Factor(EGF), Eotaxin/CCL11, Fibroblast Growth Factor (FGF-2), Fms-related tyrosine kinase 3 ligand (Flt-3 ligand), Fractalkine, Granulocyte colony stimulating factor(G-CSF), Granulocyte Macrophage Colony Stimulating Factor(GM-CSF), CXCL1/GRO, Interferon- α2(IFN-α2), Interferon-γ (IFN-γ), Interleukin-1α(IL-1α), Interleukin-1β(IL-1β), Interleukin-1receptor antagonist(IL-1ra), Interleukin-2(IL-2), Interleukin-3(IL-3), Interleukin-4(IL-4), Interleukin-5(IL-5), Interleukin-6(IL-6), Interleukin-7(IL-7), Interleukin-8(IL-8), Interleukin-9(IL-9), Interleukin-10(IL-10), Interleukin-12p40{IL-12 (p40)}, Interleukin-12p70{IL-12 (p70)}, Inteleukin-13(IL-13), Interleukin-15(IL-15), Interleukin-17A(IL-17A), Interferon- γ produced protein-10(IP-10), Monocyte Chemoattractant Protein-1(MCP-1), Monocyte Chemoattractant Protein-3(MCP-3), Macrophage Derived Chemokine{MDC(CCL22)}, Macrophage Inflammatory Protein-1α (MIP-1α), Macrophage Inflammatory Protein-1β(MIP-1β), Platelet Derived Growth Factor-AA(PDGF-AA), Platelet Derived Growth Factor-AB/BB(PDGF-AB/BB), RANTES, Transforming Growth Factor-α (TGF-α), Tumour Necrosis Factor-α(TNF-α), Tumour Necrosis Factor-β(TNF-β), Vascular Endothelial Growth Factor(VEGF). The detection limit for any analyte was 1pg/ml, with a dynamic range up to 10,000pg/ml (according to the manufacturer’s protocol). Briefly, 25ul tear sample, standards (2,000, 400, 80, 16, and 3.2 pg/mL), quality controls were incubated with antibodies-coated captured beads for 2 hours in an orbital shaker (Fischer Scientific, New Hampshire, United states) at 750 rpm, RT. Washed beads were further incubated with biotin-labelled detection antibodies for 1 hour, followed by 25ul of streptavidin-phycoerythrin incubation for 30 minutes. After washing plate, beads were resuspended in 150ul of sheath fluid for 5 minutes. The concentrations of human cytokines in tear samples were measured using Luminex 200^TM^ (Luminex Corporation, Texas, USA) instrument, the software used was xPONENT software. Standard 5 parameter logistic curve plots of known concentrations of recombinant human cytokines were used to convert Median Fluorescence Intensity (MFI) units to cytokine concentration (pg/ml).

### Statistical methods

Analysis was done using Stata Corp (version 10, USA) and GraphPad Prism (Version 7, California, USA). Quantitative data were represented as Mean (±SD) while categorical data were represented by frequency or proportions. Difference in clinical variables were analyzed using Student “t” test for comparing between eyes treated with latanoprost and bimatopost (and One-way Anova while comparing eyes treated with both PGA’s with controls) with significance set at p<0.05 The pearson’s correlation/linear regression tests was done for the correlation of different cytokines/chemokines between the groups.

## Results

We obtained tear samples from 30 eyes in each group which include primary open angle glaucoma (n = 26 and n’ = 20), POAG; normal tension glaucoma NTG; (n = 4 and n’ = 10), in latanoprost and bimatoprost group respectively, with a mean age of 62±10.5 years. The mean duration of use of PGA in both groups did not differ significantly, p = 0.6, Table 1. The two groups did not differ significantly in the baseline extent of damage at presentation, sex or IOP at presentation, Table 1.

**Table 1:** Baseline clinical characteristics of patients with glaucoma on topical latanoprost (Xalatan 0.005%) or bimatoprost (Lumigan 0.01%).

Variables	Bimatoprost N = 30	Latanoprost N = 30	Control N = 30
Age (years)	62.8±10.5	62.1±10.1	61.21±12.2
M:F (%)	53:47	72:28	73.91:26.08
Mean Deviation (dB)	-8±2.1	-9±3.2	-0.2±0.8
Pattern standard deviation (dB)	10±3.6	12±2.3	2±1.8
Baseline IOP (mm Hg)	24±2.4	20±3.8	12±1.3
Duration of prostaglandin use	1.3 (0.5–4)	1.1(0.5–5)	NA

NA: Not Applicable

The clinical side effects was marginally greater with bimatoprost though the types of ocular side effects in both groups ranged from mild hyperemia to severe burning and redness which was however no significantly different in both groups, Table 2. No patient experienced any systemic side effects. One patient had discontinued bimatoprost on night before the day of tear collection due to hyperemia on self-discretion with no evident allergic signs in the eye which mandated switch to alternate class of anti-glaucoma drug.

**Table 2:** Clinical side effects seen in patients with primary glaucoma on latanoprost 0.005% or bimatoprost 0.01%.

Variables	Bimatoprost N = 30	Latanoprost N = 30
Stinging sensation on instillation	22	17
Conjunctival hyperemia	13	9
Allergic conjunctivitis	0	0
Allergic blepharitis	0	0
Conjunctival/lid pigmentation	2	0
Others	Elongate lashes-7	Transient Drowsiness-1

### MMPs profile in latanoprost and bimatoprost treated eyes

**Fig 1:**
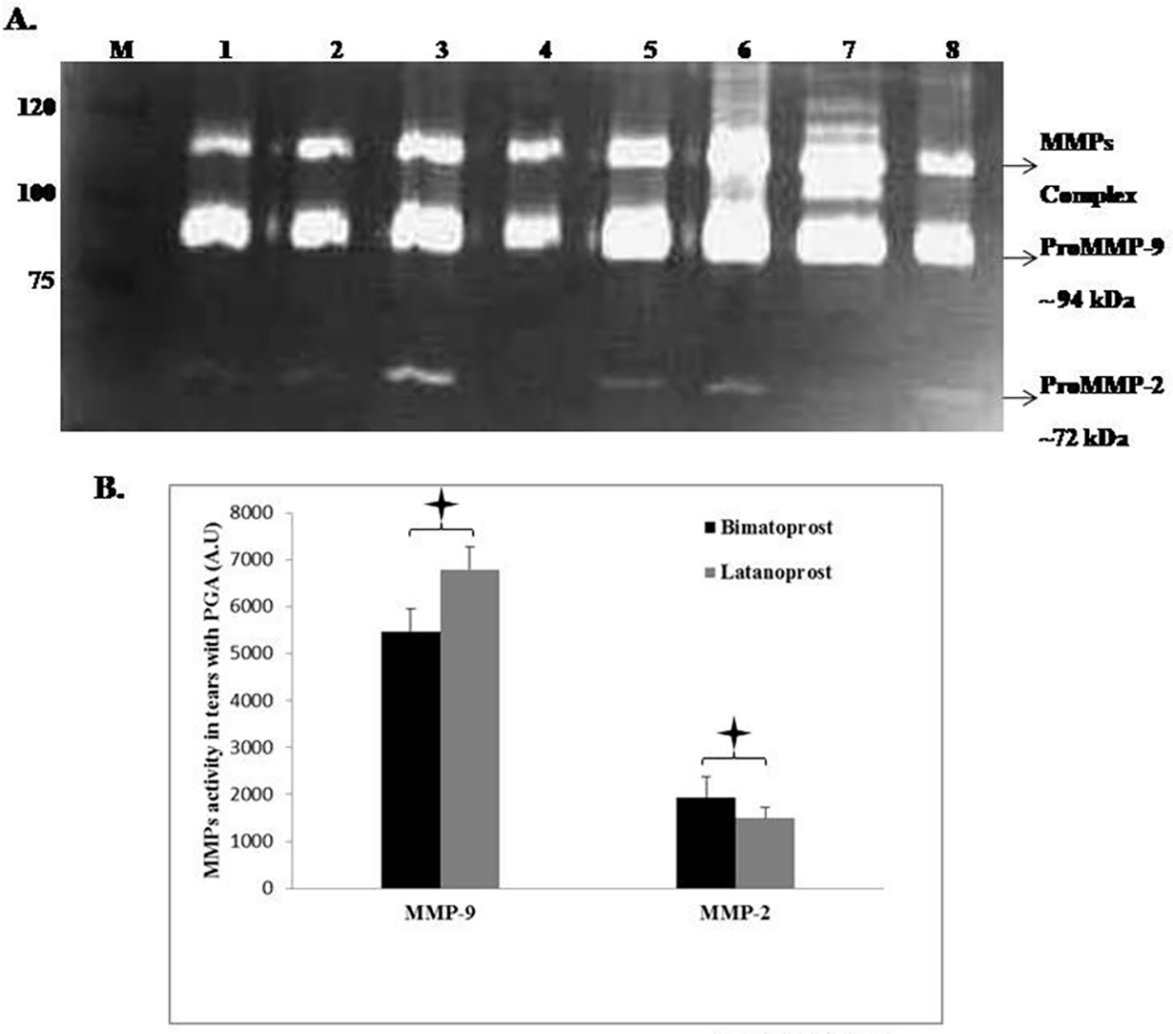
Expression of MMP-2 and **MMP-9** in tear samples of patients treated with laatanoprost or bimtorpost (n = 30 each):**A.** Representative image showing expression of MMP-2 and MMP-9 by gelatine zymography. M: Protein molecular weight Marker, Lane 1–4: Bimatoprost, Lane 5–8: Latanoprost. **B.** Densitometric analysis of gelatine zymographic gels showing overexpression of MMP-9 in latanoprost treated eyes with lower expression of MMP-2 (star indicates statistically significant difference on unpaired “t” test between the two groups). Error bars indicates the standard error of mean. A.U- Arbitrary unit. Here 50 μg/ml of total protein was loaded for each sample in the well.

The MMP-9 expression was higher in eyes receiving latanoprost while the MMP-2 expression was higher in eyes receiving bimatoprost. Moreover, both the proteases show higher expression in the drug treated groups than the control group, Fig 1, Table A in S1 File, S1 Fig.

**Fig 2:**
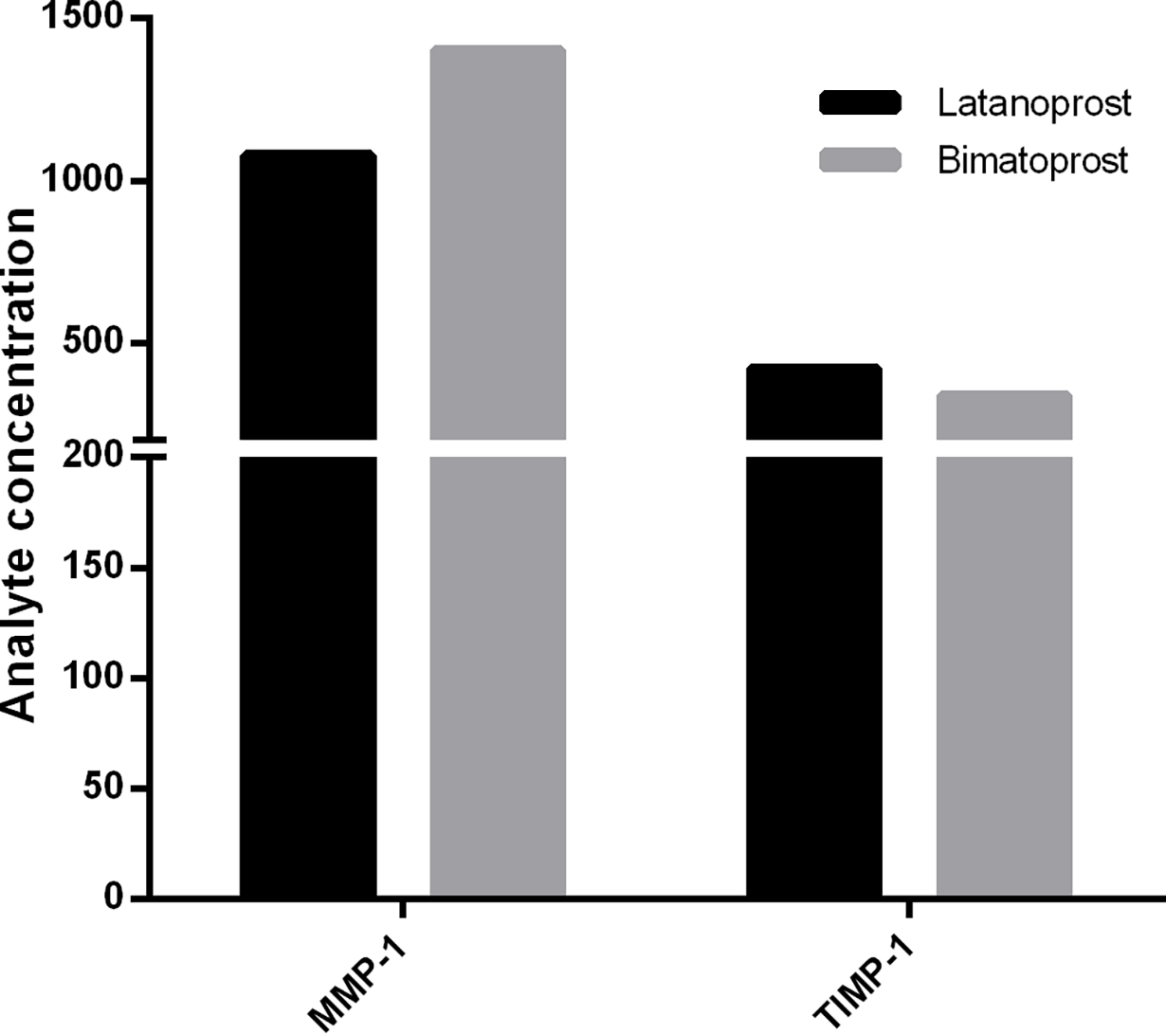
Concentration of MMP-1 (pg/ml) and TIMP-1 (ng/ml) in pooled tears samples of eyes treated with latanoprost 0.005% or bimatoprost 0.01% (n = 30).

The proteins levels of MMP-1 and TIMP-1 were determined by respective ELISAs in the extracted tears sample with PGAs. The amount of MMP-1 was found to be higher (23.1% percentage difference) in eyes with bimatoprost compared to latanoprost whereas the amount of TIMP-1 was found to be higher (by 19.1%) in eyes treated with latanoprost (Fig 2). Interestingly, level of MMP-1 protein was seen to be higher in tear samples of cataract patients when compared with the latanoprost treated group. Also a distinctive difference in TIMP-1 amount was not seen while comparing the control group versus bimatoprost treated group of patients, Fig 2, Tables A and B in S1 File

### Multiplex cytokines

**Fig 3:**
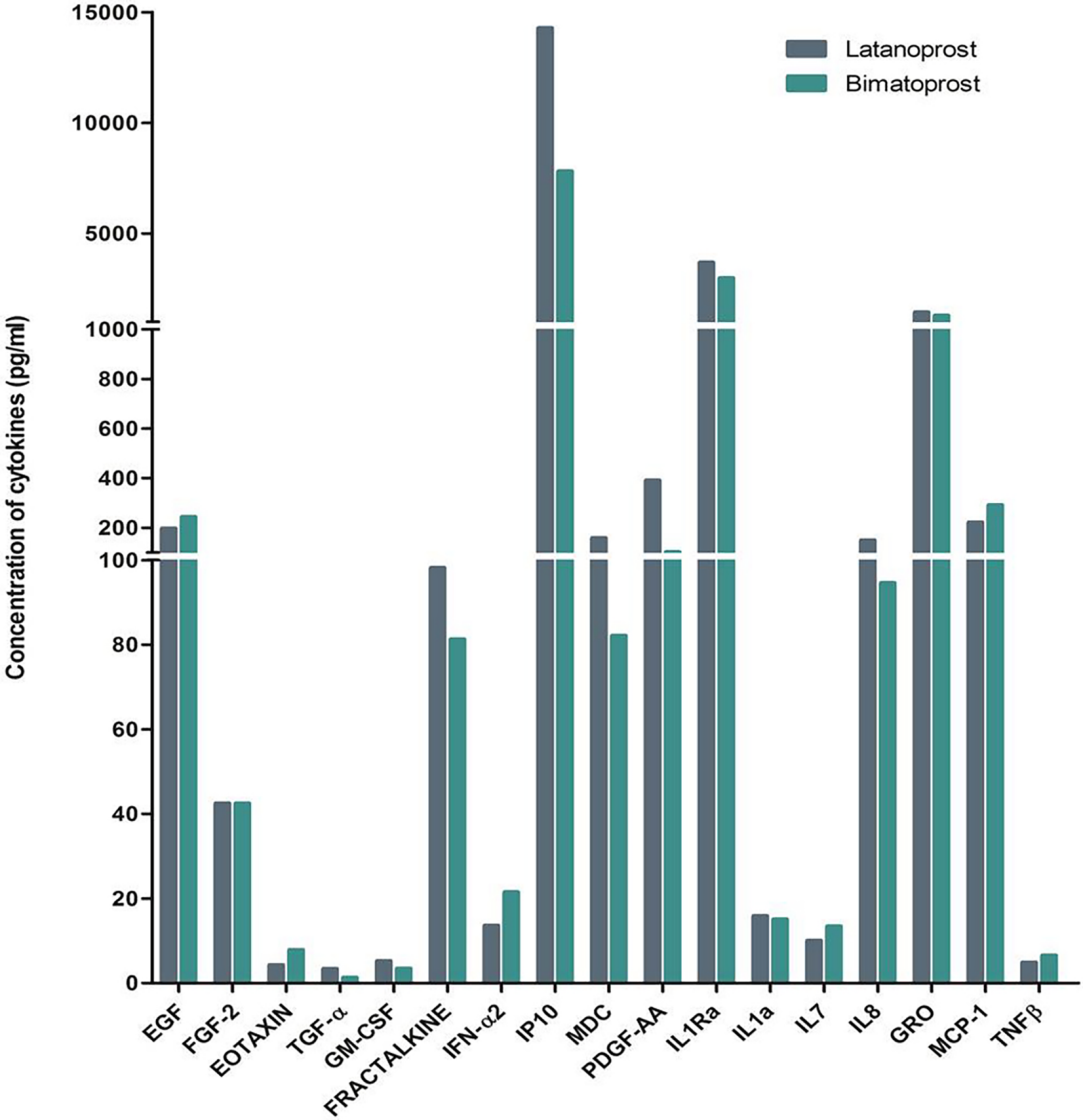
Level of expression of cytokines in pooled samples of tears of patients with primary glaucoma treated with latanoprost 0.005% and bimatoprost 0.01% (n = 20 each) showing overexpression of tissue remodeling cytokines in latanoprost treated eyes compared to bimatoprost treated eyes (see text for description).

The 41 plex multiplexed bead assay categorized a differential expression profile of the cytokines in both groups, Fig 3. The latanoprost treated eyes expressed higher levels of tissue remodelling cytokines like Granulocyte Macrophage Colony Stimulating Factor (GM-CSF), Fractalkine, Interferon-ɣ induced protein-10 (IP-10), Macrophage Derived Chemokine (MDC), Platelet Derived Growth Factor-AA (PDGF-AA), Interleukin-1a (IL-1a), Interleukin 1 receptor antagonist (IL-1Ra), Interleukin 8 (IL-8), Chemokine CXCL-1 with fold changes of 1.5, 1.2, 1.8, 1.9, 3.8, 1.2, 1.6, 1.1 respectively compared to bimatoprost. Epidermal Growth Factor(EGF), Eotaxin, Interferon-α2 (IFN-α2), Interleukin-7 (IL-7), Monocyte Chemoattractant Protein-1 (MCP-1), Tumour Necrosis Factor- β (TNF-β) were elevated in the bimatoprost treated group with respective fold change of 1.2, 1.9, 1.6, 1.3, 1.3, 1.3 respectively, majority of which were related to allergic effects. However, Fibroblast Growth Factor-2 (FGF-2) expression found in wound healing processes which was equal in both the test group (42.45pg/ml). It is intriguing to see the increased levels of Interferon-ɣ induced protein-10 (IP-10), Macrophage Derived Chemokine (MDC), Platelet Derived Growth Factor-AA (PDGF-AA), Interferon-α2 (IFN-α2), Interleukin-1a (IL-1a), Interleukin 1 receptor antagonist (IL-1Ra), Interleukin 8 (IL-8), Interleukin 7 (IL-7), Chemokine CXCL-1 Tumour Necrosis Factor- β (TNF-β), Fibroblast Growth Factor-2 (FGF-2) on comparing PGA average versus the control cytokine concentrations, S2 Fig.

### Correlation of cytokines/chemokines between PGAs treated groups

**Fig 4:**
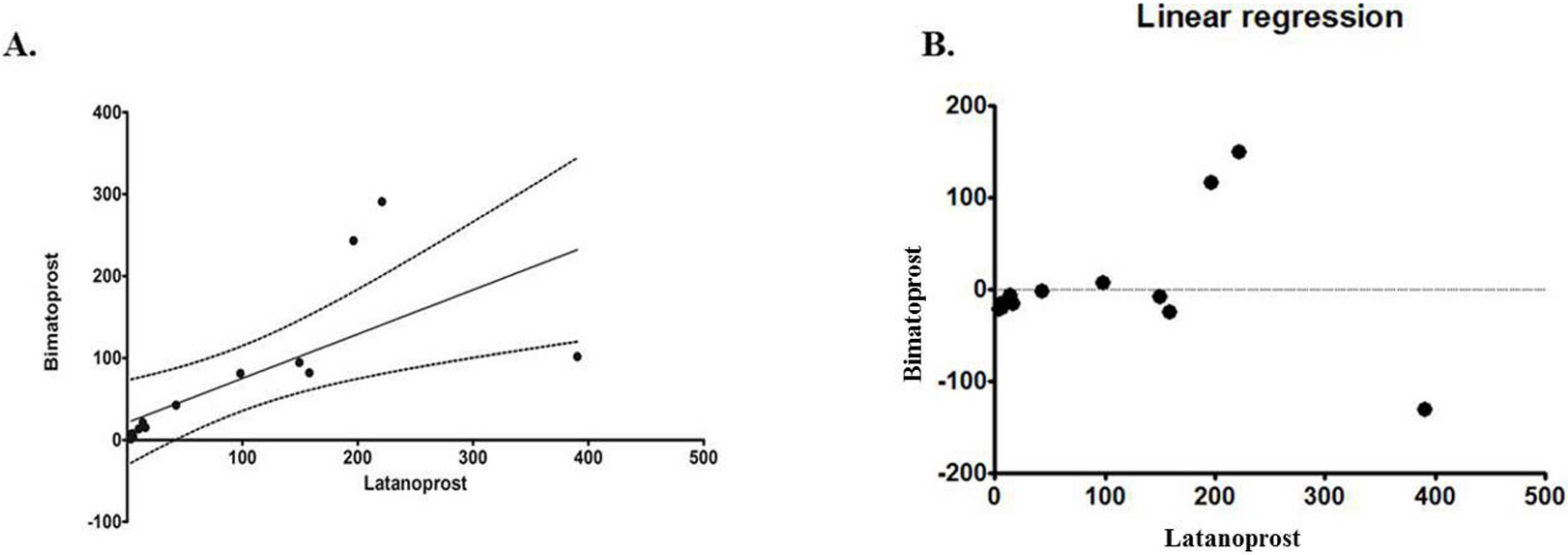
Correlation analysis of cytokines/chemokine levels in eyes with primary glaucoma treated with latanoprost or bimatoprost (n = 20 each) showing positive correlation of cytokine levels between the two groups.

Correlation computation helps assess the linear association between the two continuous variable drug treated groups consisting concentration of cytokines analysed. The correlation of different cytokines/chemokines among PGA treated groups was done using pearson’s correlation test. Among 14 different cytokines with the range of 1–500 pg/ml (EGF, FGF-2, eotaxin, TGF-a, GM-CSF, fractalkine, IFN-a2, MDC, PDGF-AA, IL1a, IL7, IL8, MCP-1, TNF-b), FGF-2, fractalkine, IL8 showed a significant positive correlation between the groups, r = 0.96, p<0.0001, (Fig 4).

The cytokine levels did not correlate among the groups for different clinical variables like age, IOP or duration of medication use. S2 Fig; S3 Fig; S4 Fig respectively.

## Discussion

This study highlights an elevated MMP-9 expression in the latanoprost treated group with increased MMP-2 expression in the bimatoprost (0.01%) treated group. This was paralleled with similar decrease in TIMP-1 levels though MMP-1levels did not vary significantly in the two groups. These suggest definite differential changes in tear MMP levels in regulating matrix degradatory process in bimatoprost and latanoprost groups. This was also seen by the cytokine analysis which showed predominance of pro-inflammatory cytokines in the latanoprost treated eyes.

MMP’s are key molecules involved in extracellular matrix homeostasis which are regulated by tissue inhibitors like TIMP-1.[3,6,7,12–14] These molecules are expressed in response to inflammatory stimulus or insult at the ocular surface like use of anti-glaucoma drops.[] Inflammation is known to induce expression of MMP and inflammatory cytokines which are predominant mechanism of balancing ECM formation and degradation.[7] The results of this study suggest greater upregulation of pro-inflammatory cytokines in latanoprost which contrasts with earlier reported greater clinical hyperemia with bimatoprost 0.03%. This is concordant with earlier studies showing greater inflammation with PGA’s though this study also showed significantly greater cytokine expression with even bimatoprost concentrations of 0.01% used in this study. This may possibly the reason for mildly greater predominance of pro-inflammatory cytokines with latanoprost in contrast to earlier studies reporting greater inflammation with bimatoprost 0.03%.[9–12]

Previous studies have shown increased MMP expression after PGA’s on keratocytes and epithelial cells in-vitro.[8,11,12,13,15–25] This family of proteins have been shown to regulate a wide variety of physiological & pathological processes, including wound healing, inflammation, tissue remodelling, growth and development apart from eyes on topical anti-glaucoma medications.[7,13,14–16] Specifically, MMP-2 and MMP-9 are found in tears during ocular surface disease, including dry eye and keratoconus.[7,14–16] These enzyme isoforms are regulated by endogenous tissue inhibitors of metalloproteases (TIMPs) which are key in maintaining MMP regulation and ECM degradation.[7]

MMPs may be up-regulated in glaucoma per se which in essence constitutes an ECM disorder.[13] Further, these mechanisms may be differently regulated at different stages of glaucoma irrespective of the effect of external agents like glaucoma medications.[18] Yet, we compared patients with similar stages of glaucoma who were PG analogues for a similar period of six months; so effect of stage of glaucoma may be smoothened out by choosing all eyes with similar stage of glaucoma for specified period of medications. Preservatives like BAK also have been reported have inflammatory effect secondary to long term use of glaucoma medications.[8,19,20] Yet, this study with the same preservative found significant difference in tear MMP profiles suggesting differential level of activation of inflammatory pathways owing to different receptors involved in mechanism of drug action.

Latanoprost treatment increased levels of MMP9 while bimatoprost treated eyes had increased levels of MMP2 and MMP1. Strong significant pearson’s correlation was seen between the groups for FGF-2, fractalkine, IL8 among all other cytokines which are involved in tissue remodelling with IL8 also involved in mediating tissue fibrosis by macrophage recruitment. While all MMP’s are controlled at transcriptional level, MMP2 is constitutively expressed and MMP1 expression or repression is dependent on substrate (collagen 1) availability.[7,13] It may be possible that latanoprost induces more constitutional tissue expression of MMP2 in the ocular surface while bimatoprost induces predominant inflammatory cytokine response through independent receptors and signalling pathways as seen in the this study. This is in concordance with clinical hyperemia being more with use of bimatoprost.[2,3,12] While Eotaxin-1 is an eosinophil chemoattractant which has been found to correlate with PGE2 levels in proliferative diabetic retinopathy and proliferative vitreoretinopathy (PVR), MCP-1 is a monocyte recruiting chemokine which has been elevated in eyes post LASIK ectasia and allergic conjunctivitis.[26–29] This is in concordance with similar results in other conditions like proliferative diabetic retinopathy, vernal kertoconjunctivitis.[28] Cytokines like PDGF-AA and MDC have been found to be upregulated in dry eye and wet age related macular degeneration.[27] Other pro-inflammatory cytokine like IP10, a macrophage attractant, were also high in latanoprost treated eyes.[5,7,29] It may be possible that the local tissue modelling effects are mediated by these molecules which may be higher with latanoprost inducing more of constitutional MMP or cytokine response than bimatoprost mediating predominant generalised inflammatory response. Yet, we used pooled tear samples for cytokine analysis and therefore these results cannot be directly generalised.

Topical prostaglandins have been shown to cause activation of MMPs and subclinical inflammation in the conjunctiva and ocular surface accounting for frequent side effects associated with long term use of these agents.[8–12, 15–26] An earlier study has reported increased levels of MMP1 and reduced TIMP1 levels after latanoprost in tears and conjunctival surface in mouse compared to controls.[25] Topical PGA’s are known to induce inflammation thereby limiting their use in inflammatory cases (like uveitis, cystoid macular edema and post-surgery)and the effect is reported to be more with bimatoprost acting on prostanoid receptors.[2,3] Attempts at reducing this inflammatory hyperemia and side effects include reducing concentrations, altering or removing the preservative and also changing manufacturing processes. Bimatoprost 0.01% is the result of such endeavours to reduce hyperemia with significantly reduced side effects like dry eye, stinging, burning or hyperaemia.[9] Though clinical side effects have been proven to be less as compared to 0.03%, laboratory validation of such a clinical effect is scarce. This study suggests that the inflammatory pathways would still be up-regulated with Bimatoprost 0.01% albeit at lower levels compared to latanoprost. Further research is required to investigate if latanoprost and bimatoprost regulate MMP differentially via the TGF signalling pathway.

In summary, latanoprost treated eyes had overexpression of MMP9 along with other inflammatory cytokines related to tissue remodelling while bimatorpost treated eyes had increased cytokines primarily related to allergic responses in the eye implying differential mechanism of inflammatory modulation by these two PGA’s in glaucoma. These can be explored to minimise ocular side effects seen with both drugs used commonly in glaucoma patients.

We did not evaluate the actual tissue levels of PGE2 and compare them with different cytokine levels in this study. We also did not recruit patients on other glaucoma drugs to first evaluate differential expression with PG group of drugs in the eye. Also we did not study the tissue related effect on the ocular surface or tissue based markers indicating upregulated inflammation or fibroblast activation. However we find similar expression levels of Fibroblast Growth Factor (FGF-2) responsible in wound healing processes. Nevertheless, differential cytokine expression between latanoprost and bimatoprost signal differences in mechanism of action via different regulatory pathways which needs further evaluation.

## Supporting information

S1 Fig Tear cytokines profile in primary glaucoma eyes treated with latanoprost or bimatoprost versus controls (cataract patients). (TIF)

S2 Fig Association of tear cytokine concentrations in eyes treated with PG Analogs like latanoprost and bimatoprost with the age of the patient. (TIF)

S3 Fig Association of mean tear cytokine concentrations in eyes treated with PG Analogs like latanoprost and bimatoprost with IOP (Intra Ocular Pressure). (TIF)

S4 Fig Association of cytokine concentrations of PG Analogs individually with mean duration of medication use. (TIF)

S1 File Supplemental Tables A and B.
(DOCX)

S1 Data Supporting raw excel file containing minimal data of the study. (XLSX)
