# Supplementary figures and images for: Tear biomarkers in latanoprost and bimatoprost treated eyes
Source: PLoS One. 2018 Aug 6;13(8):e0201740. doi: 10.1371/journal.pone.0201740 (PMC6078293; doi:10.1371/journal.pone.0201740)

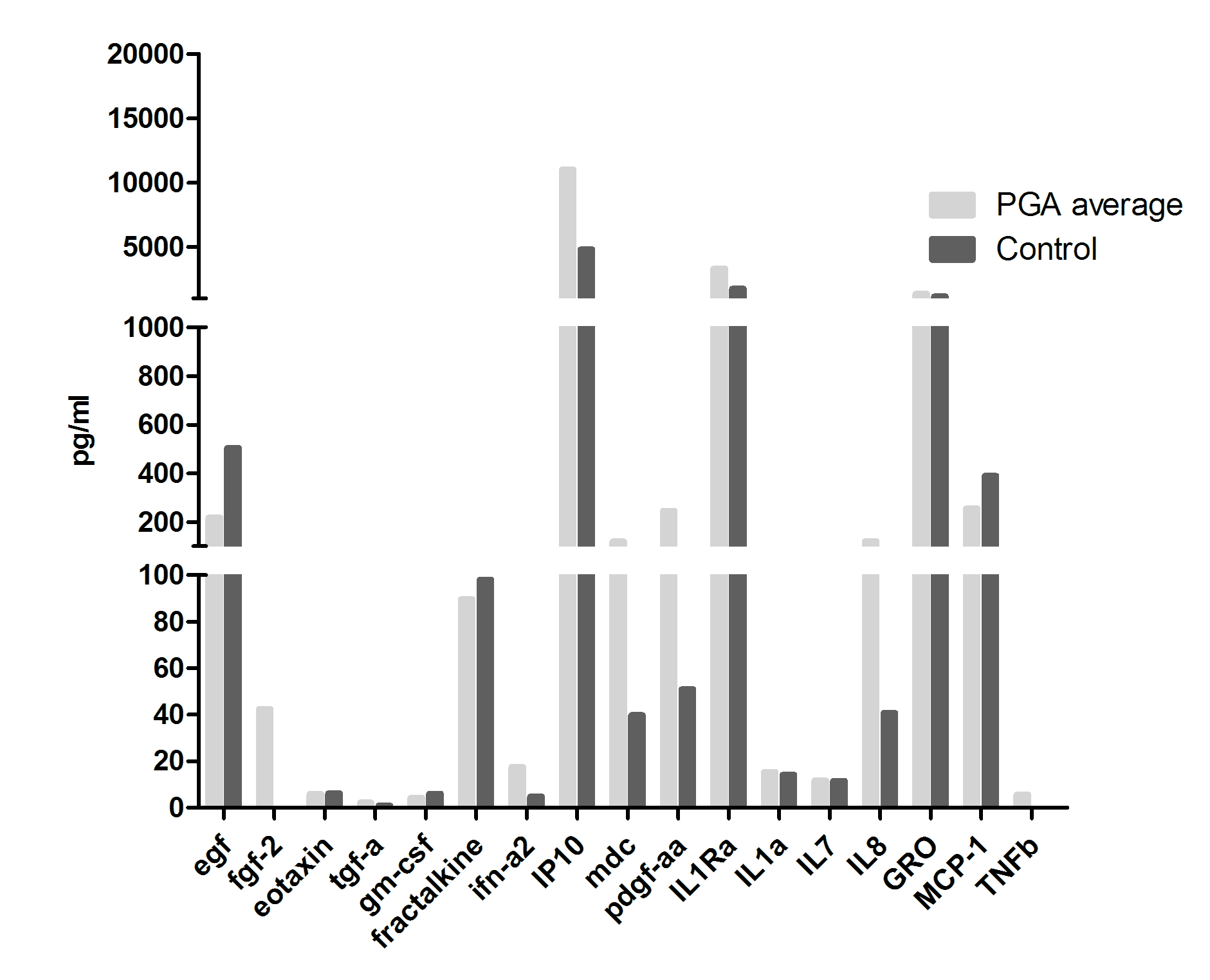

Supplement: S1 Fig — (TIF) [file pone.0201740.s001.tif]

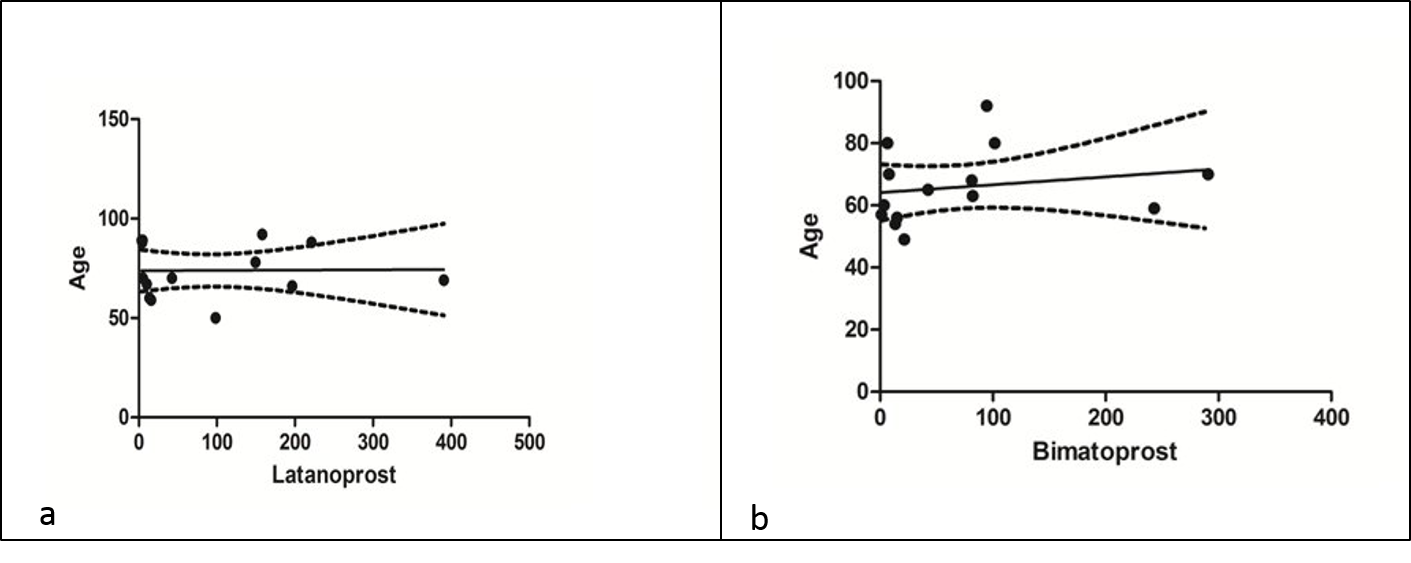

Supplement: S2 Fig — (TIF) [file pone.0201740.s002.tif]

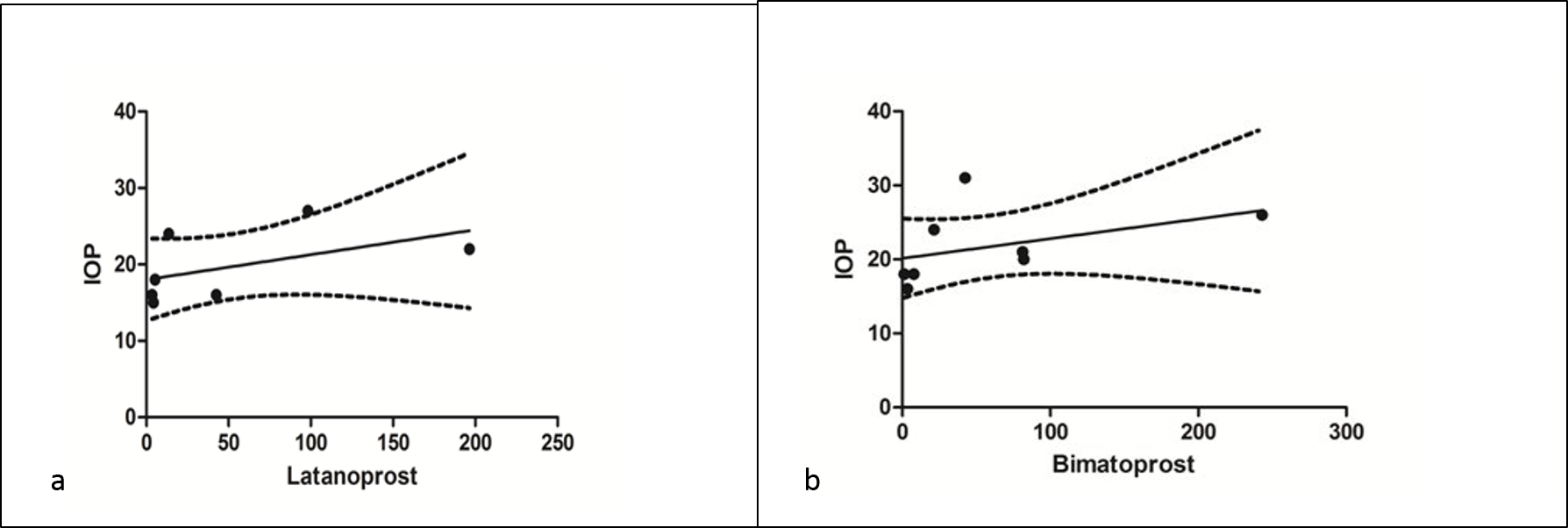

Supplement: S3 Fig — (TIF) [file pone.0201740.s003.tif]

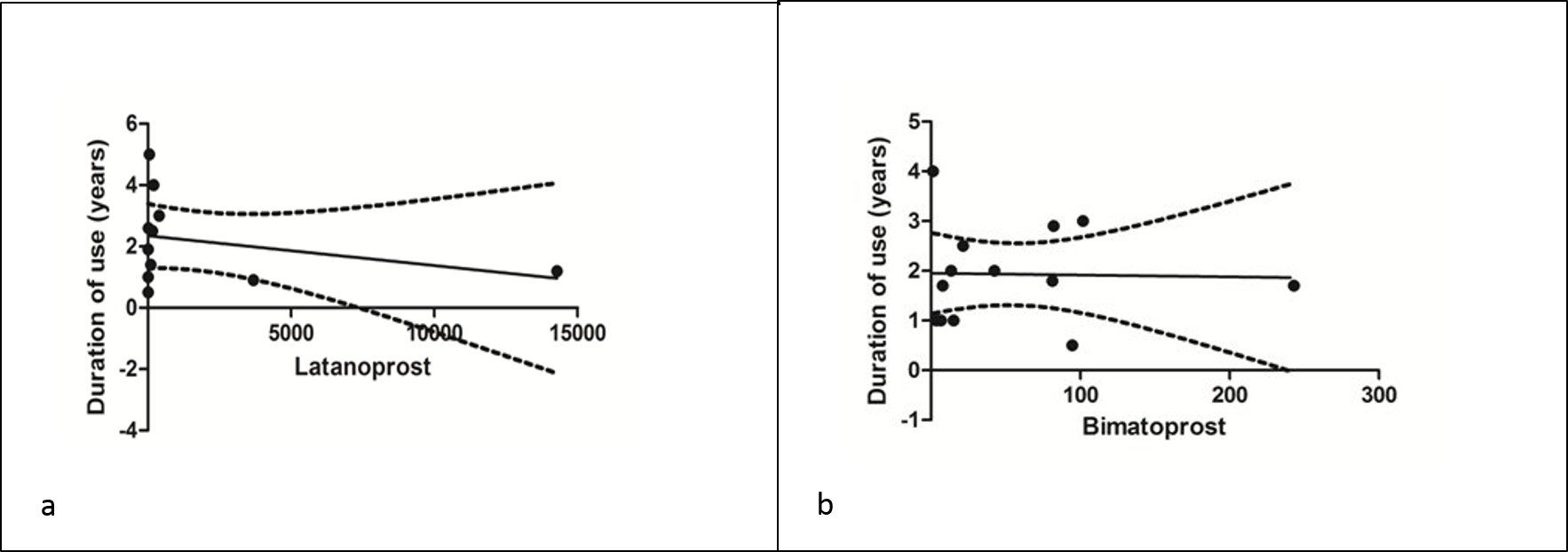

Supplement: S4 Fig — (TIF) [file pone.0201740.s004.tif]
