# Supplementary material for: Tear biomarkers in latanoprost and bimatoprost treated eyes
Source: PLoS One. 2018 Aug 6;13(8):e0201740. doi: 10.1371/journal.pone.0201740 (PMC6078293; doi:10.1371/journal.pone.0201740)
Supplement: S1 File — Supplemental Tables A and B. (DOCX) [file pone.0201740.s005.docx]

**Table A. Expression of MMP-9 and MMP-2 in tears samples of patients with patients treated with (latanoprost 0.005% or bimatoprost 0.01%) and controls by gelatin zymography.**

| **MMPs** | **Bimatoprost**  **(mean±SEM)**  **( n=30)** | **Latanoprost**  **(mean±SEM)**  **(n=30)** | **Control**  **(mean±SEM)**  **(n=30)** | **P-value** |
| --- | --- | --- | --- | --- |
| **MMP-9**  **(**A.U) | 5458.06±491.38 | 6795.78±491.21 | 950.66±160.69 | <0.0001^#^ |
| **MMP-2**  **(**A.U) | 1939.04±439.41 | 1497.76±223.12 | 578.51±111.33 | <0.0001^#^ |

. (A.U): Arbituary Unit, ^#^-One-way Anova between all three groups.

**Table B: Tear expression of MMP1 and TIMP1 in primary glaucoma among prostaglandin treated eyes (latanoprost 0.005% or bimatoprost 0.01%) and controls.**

| **MMPs** | **Bimatoprost**  **(n=10)** | **Latanoprost**  **(n=10)** | **Control**  **(n=10)** | ***P* Value** |
| --- | --- | --- | --- | --- |
| **Concentration of MMP-1 (pg/ml)** | 1404 | 1079 | 1304 | 0.04^#^ |
| **Concentration of TIMP-1 (ng/ml)** | 339.03 | 420.8 | 333.45 | 0.02^#^ |

^#^ -One-Way Anova between all three groups
